# Small Fiber Neuropathy Associated with Post-COVID-19 and Post-COVID-19 Vaccination Arthritis: A Rare Post-Infective Syndrome or a New-Onset Disease?

**DOI:** 10.3390/jpm14080789

**Published:** 2024-07-25

**Authors:** Francesca Bandinelli, Romina Nassini, Eleonora Gherardi, Barbara Chiocchetti, Mirko Manetti, Massimo Cincotta, Filippo Nozzoli, Elena Nucci, Francesco De Logu, Nicola Pimpinelli

**Affiliations:** 1 Rheumatology Department, San Giovanni di Dio Hospital, Usl Tuscany Center, 50143 Florence, Italy; 2 Section of Clinical Pharmacology and Oncology, Department of Health Sciences, University of Florence, 50139 Florence, Italy; romina.nassini@unifi.it (R.N.);; 3 Headache Center and Clinical Pharmacology Unit, Careggi University Hospital, 50139 Florence, Italy; 4 Section of Dermatology, Department of Health Sciences, University of Florence, 50125 Florence, Italynicola.pimpinelli@unifi.it (N.P.); 5 Neurology Department, San Giovanni di Dio Hospital, Usl Tuscany Center, 50143 Florence, Italymassimo.cincotta@uslcentro.toscana.it (M.C.); 6 Section of Anatomy and Histology, Department of Experimental and Clinical Medicine, University of Florence, 50134 Florence, Italy; mirko.manetti@unifi.it; 7 Section of Pathological Anatomy, Department of Health Sciences, University of Florence, 50139 Florence, Italy; 8 Histopathology and Molecular Diagnostics Unit, Careggi University Hospital, University of Florence, 50139 Florence, Italy

**Keywords:** small fiber neuropathy, COVID-19, arthritis

## Abstract

Post-COVID-19 (PC) and post-COVID-19 vaccination (PCV) syndromes are considered emergent multidisciplinary disorders. PC/PCV small fiber neuropathy (SFN) was rarely described and its association with undifferentiated arthritis (UA) was never defined. We aimed to evaluate PC/PCV-UA associated with the recent onset of severe lower limb paresthesia, compare SFN positive (+) to negative (−) patients, and evaluate changes in biomarkers in SFN+ during treatments. Nineteen PC/PCV-UA-patients with possible SFN underwent skin biopsy at the Usl Tuscany Center (Florence) early arthritis outpatient clinic from September 2021 to March 2024. Eight selected SFN+ were compared to ten SFN− patients. In SFN+ patients, baseline joint ultrasound (US), electromyography (EMG), optical coherence tomography (OCT), and skin biopsy were repeated at six months. Moreover, SFN+ patients were clinically assessed by a 0–10 numeric rating scale for neurological symptoms and DAS28/ESR up to 12 months follow-up. SFN+ patients showed a lower intraepidermal nerve fiber density at histopathological examination of skin biopsies and a higher frequency of OCT and EMG abnormalities in comparison to SFN− patients. In SFN+ patients, US and DAS28/ESR significantly improved, while intraepidermal nerve fiber density did not significantly change at the six-month follow-up. Fatigue, motor impairment, burning pain, brain fog, and sensitivity disorders decreased at long-term follow-up (12 months).

## 1. Introduction

Coronavirus disease 2019 (COVID-19), a newly emerging infectious disease caused by a novel coronavirus termed severe acute respiratory syndrome coronavirus 2 (SARS-CoV-2), has become a global pandemic burden. The disease was predominantly characterized by lung damage and hypoxia resulting in systemic complications, and even death. However, following the acute infection, many patients reported long-term effects characterized by a wide range of symptoms and complications extending far beyond the initial respiratory symptoms, which have been referred to as long-term COVID-19 [1]. These symptoms are different, involving multiple organ systems such as respiratory distress (cough and dyspnea), muscle pain, fatigue, headache, taste or smell impairment, and brain fog [2].

In addition to long COVID-19 symptoms, several patients report neurological complications following COVID-19 vaccination mainly related to vascular, immune, infectious, and functional factors [3]. These conditions may present as a recurrence of previous neurological syndromes or new-onset disorders [3]. Nonetheless, the frequency of occurrence, as well as the varying host and vaccine features, clinical presentations, treatment approaches, and outlooks vary considerably. While the reason for the variety of rare long-term post-COVID-19 (PC) and post-COVID-19 vaccination (PCV) consequences is not well understood, molecular mimicry between human proteins or other human pathogens and spike antigenic viral proteins are the most feasible explanation for these unexpected interactions with host cells [4,5]. One important complication of PC and PCV disorders is the development of peripheral neuropathy, a condition that results from damage to the peripheral nervous system, including nerves responsible for transmitting sensory information, motor function, and autonomic functions like heart rate and digestion [6].

Neuropathic pain may stem from abnormalities in the somatosensory system, particularly affecting small nerve fibers. These alterations can be identified through a few exploratory and pioneering instrumental investigations [7,8], but not with electromyography (EMG) which is commonly used in routine clinical practice. To date, skin histological examination represents the gold standard for the diagnosis of small fiber neuropathy (SFN) that can manifest with common symptoms including tingling sensations, burning pain, numbness, muscle weakness, and loss of coordination. These symptoms can affect the limbs, hands, and feet, resembling other neuropathic conditions. Fatigue and lower limb paresthesia are frequently initial, confounding, and often self-limiting manifestations in the PC and PCV period, and, for this reason, they are frequently underestimated [9].

In addition, a greater incidence of undifferentiated arthritis (UA) after SARS-CoV-2 infection was recently reported in 177 Italian patients presenting with rheumatological complications during routine clinical practice [10]. Of note, such UA condition was characterized by a rapid and acute onset with progression to early arthritis in 77% of patients. Meanwhile, polymyalgia rheumatica (PR) and Horton arteritis (HA) emerged as common adverse events of the BNT162b2 vaccine, with an elevated percentage of remission after six months [10]. Consistent with these observations, recently published systematic reviews including case reports and case series from different parts of the world showed that the most common rheumatic diseases following PCV were UA, PR, and HA [11]. Finally, PR-like inflammatory muscle symptoms and myositis [10], myocarditis [12], and vasculitis [13] have also been described.

At present, neuropathic-like pain symptoms have been reported in the early stages of rheumatoid arthritis and were found to represent unfavorable prognostic factors to achieve short-term remission [14,15]. Conversely, to the best of our knowledge, SFN neuropathic pain associated with UA in PC and PCV syndromes has not yet been described.

On these premises, the current study was undertaken to examine the clinical features and biomarkers of skin biopsy-diagnosed SFN-positive (+) patients with UA developed after PC or PCV syndromes, in comparison to SFN-negative (−) patients with similar new-onset PC- and PCV-associated lower limb paresthesia. Furthermore, SFN+ cases were evaluated at long-term follow-up for disease persistence and evolution during treatment.

## 2. Materials and Methods

### 2.1. Study Design and Patients

We retrospectively studied patients presenting with PC and PCV rheumatological complications of early UA diagnosed with EULAR/ACR 2010 criteria [16] associated with new-onset severe lower limb paresthesia within 4 weeks after infection or vaccination, and not previously treated with steroids or disease-modifying antirheumatic drugs (DMARDs). Patients were referred to the early arthritis outpatient clinic of the San Giovanni di Dio Hospital of Florence (Italy) by general practitioners and followed up in Day Service at the outpatient clinic by an expert rheumatologist (FB) according to regional guidelines and Italian Health Ministry legislation (22 April 2021, no. 52) from September 2021 to March 2024. After preliminary neurological clinical and EMG evaluations excluding large fiber neuropathy, patients with severe lower limb sensory symptoms suggestive of SFN (i.e., thermal sensitivity disarray, burning and shooting pain at distal parts, prickling, numbness, or tightness with stocking/glove-like perception) were referred to the Section of Dermatology of the University of Florence, where they underwent skin biopsy (T1, baseline). Concomitant diabetes, active infections, and tumors were excluded. Moreover, no patient had previous or concomitant relevant neurological or rheumatological disorders, such as fibromyalgia, before the occurrence of suggestive symptoms for SFN after PC and PCV. Patients with histological evidence of SFN (SFN+) were successively followed up by a rheumatologist (FB) and a neurologist (BC), as well as by a dermatologist (EG) who performed a second skin biopsy six months after the first evaluation (T2, six-month follow-up). Patients without histological evidence of SFN (SFN−) also underwent follow-up, as well as a second skin biopsy after six months only in case of persistence or progression of neurological symptoms. All SFN+ patients at T2 were further followed up at 9 months (T3) and 12 months (T4) with both rheumatological and neurological examinations. Demographics and clinical features and laboratory and instrumental examination data at baseline and follow-up were saved in ARGOS Usl Tuscany Center electronic chart multidisciplinary files. Privacy consent for anonymous analysis and publication of routine clinical data was given by each patient and saved in the Argos electronic chart of Usl Tuscany center, as per the Declaration of Helsinki on investigation in humans and according to the Tuscany Region Institutional Review Board resolution (no. 450) and Italian legislation (authorization no. 9, 12 December 2013). Only COVID-19 vaccinations approved in Italy (i.e., BNT162b2, mRNA-1273, AZD1222, and Ad26.COV2.S) were considered for PCV manifestations in the present study.

### 2.2. Measurements

Two 3-mm punch skin biopsies were performed at the distal leg (10 cm above the lateral malleolar region) and the proximal thigh (10 cm below the lateral region of the trochanter) of each patient after informed consent was obtained. Skin biopsy specimens were placed in 2% formaldehyde-lysine-periodate and immediately stored at 4 °C until sent to the Histopathology and Molecular Diagnostics Unit, Careggi University Hospital, Florence (Italy) according to P/14422/07 Ed. 2Rev. internal sample acceptance procedure, Standard UNI EN 9001 edition 2015. Three not continuous cryostatic sections of 50 µm thickness were processed and immunoassayed using an anti-PGP9.5 antibody for the evaluation of intraepidermal nerve fiber density (IENF/mm) as described elsewhere [17].

An initial neurological and electrophysiological assessment was performed, following the patient’s admission to the Rheumatology Department, using a 2-channel EMG device [18], and repeated at follow-up in case of not remittent neurological symptoms [19]. Based on neurological and skin biopsy evaluations, patients were defined SFN+ according to Bradford Hill criteria [20,21] and were clinically scored at each visit at baseline (T1) and 6, 9, and 12 months of follow-up (T2, T3, and T4, respectively) with 0–10 Numeric Rating Scale (NRS) for fatigue, motor impairment, burning pain, sensitivity disorders (e.g., thermal disarray, stocking-glove disorder, and numbness), brain and visual fog, and arthritic disease activity with the disease activity score in 28 joints (DAS28)/erythrocyte sedimentation rate (ESR). Shoulders, wrists, hands, and other affected joints were investigated through both longitudinal and transverse ultrasound (US) examinations performed by an experienced sonographer (FB) with a MyLab70 XVG machine (Esaote SpA, Genoa, Italy, multifrequency linear probe 12–15 MHz), with 750 Hz pulse repetition frequency and 53–55% dB gain for Power Doppler (PD) setting, at baseline (T1) and six months (T2). All digital images were saved and archived in a computer system. The intra-observer agreement in US assessment using the same machine showed good results (unweighted κ test = 0.90) as reported elsewhere [22]. The US alterations were assessed as follows: active synovitis (i.e., echogenic non-compressible intra-articular vascularization of the synovial membrane showing PD signal); active tenosynovitis or peri-tendinitis (i.e., hypoechoic thickened tissue with or without fluid within the sheath of flexor tendons or around the extensor tendons, respectively, observed in two perpendicular planes and showing PD signal); pseudo-tenosynovitis in hands (i.e., hypoechoic soft tissue surrounding the flexor tendons with an intense PD signal); and bone erosions (i.e., interruptions of the bone profile on two orthogonal scanning planes) [16].

After the first evaluation and before beginning treatments, peripheral venous blood samples were drawn, and the following analyses were carried out at the Immunology Laboratory of the Usl Tuscany Centre (Florence, Italy): ESR (mm/h; Alifax, Padoa, Italy), C-reactive protein (CRP, mg/dL; Beckman Coulter Inc., Brea, CA, USA), interleukin-6 (IL-6, pg/mL; Invitrogen, Bender MedSystem GmbH, Vienna, Austria), antinuclear antibodies (ANA) assayed by an indirect immunofluorescence method on HEp-2 cells (Euroimmun, Lübeck, Germany) according to the classification of the International Consensus on ANA Patterns (including sub-analysis of myositis blot, when titer was higher than 1:160), anti-SARS-CoV-2 IgG (nucleocapsid protein), anti-spike IgG (S1-RBD quantitative), NK count at immunophenotype, and HLADRB1* 11, C*07 haplotypes [10] assessed by inverse hybridization PCR (One Lambda, Canoga Park, CA, USA).

Optical coherence tomography (OCT) retinal scanning was conducted on both eyes; in particular, scans also centered the optic nerve head and quantified the peripapillary retinal nerve *fiber* layer (RNFL) thickness, including superior, nasal, inferior, and temporal quadrants.

Patients presenting dyspnea self-rating modified Medical Research Council (mMRC) questionnaire score (0–4) >2 (2 = subject walks slower than people of same age on the level because of breathlessness or has to stop to catch breath when walking at their own pace on the level) [23] were subjected to chest high-resolution computed tomography (HRCT) to analyze elementary lung lesions (i.e., subpleural fibrotic nodules, traction bronchiectasis, ground glass, reticulation, and honeycombing). When interstitial lung disease signs were detected at HRCT, the severity of lung dysfunction was scored as follows: normal, DLCO > 75% of predicted and up to 140%; mild, 60% to lower limit of normal; moderate, between 40% and 60%; severe, <40%. A DLCO < 60% or a DLCO < 75% in association with a forced vital capacity [FVC] < 80% of predicted were considered significant signs of restrictive lung disease at pulmonary function tests [24].

### 2.3. Statistical Analysis

The sample size for statistical analysis was calculated by taking as a reference a similar recent study [25]. The analyzed patient population achieved a statistical power of 80% with an α level of 0.05 at a β level of 0.2. Data were expressed as median and interquartile range (IQR; 25–75 percentile) for continuous variables or percentage for categorical variables. Either the Kolmogorov–Smirnov test or the Shapiro–Wilk test were employed to determine the distribution of variables. Non-normally distributed data were analyzed with the non-parametric Mann–Whitney U test. Categorical data were compared among groups using a Chi-square test, as appropriate. Values of *p* < 0.05 were considered statistically significant. All statistical analyses were carried out with GraphPad Prism 8.0 software. Data reported in the present study conform to the STROBE guidelines [26].

## 3. Results

**Figure 1:**
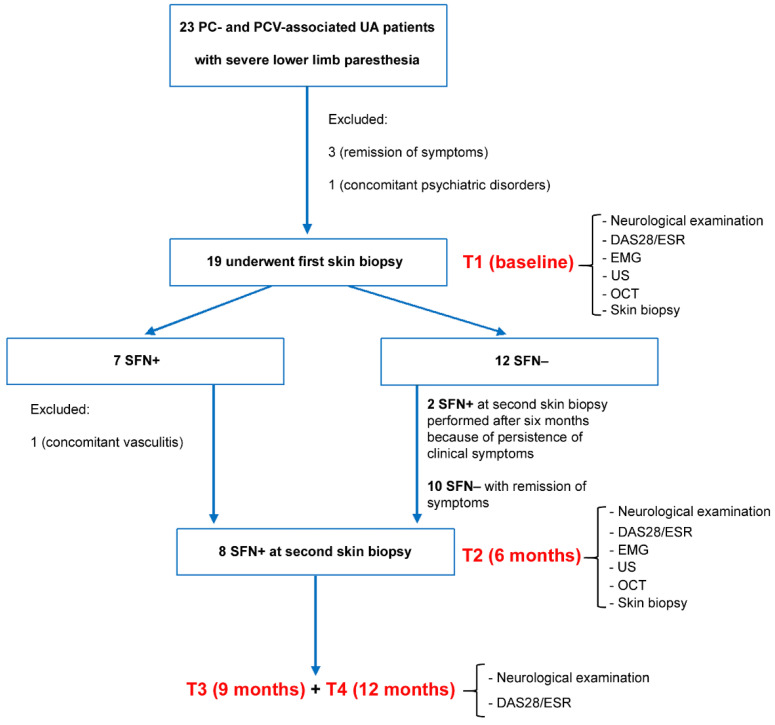
Study flow chart. Out of 23 patients with post-COVID-19 (PC) and post-COVID-19 vaccination (PCV)-associated undifferentiated arthritis (UA) with severe lower limb paresthesia, 19 underwent a first skin biopsy at baseline (T1). Seven patients resulted in small fiber neuropathy (SFN)-positive (+), while 12 were SFN-negative (−). Six SFN+ patients were confirmed and 2 SFN− patients became SFN+ at the second skin biopsy performed after six months (T2). The 8 SFN+ patients at the second skin biopsy were further followed up at 9 months (T3) and 12 months (T4) with both rheumatological and neurological examinations. DAS28/ESR, disease activity score in 28 joints calculated on erythrocyte sedimentation rate levels; EMG, electromyography; US, ultrasound; OCT, optical coherence tomography.

Twenty-three patients presenting new-onset PC- and PCV-associated UA demonstrated by the US, with a maximal interval of 4 weeks from COVID-19 infection or vaccination, were candidates for skin biopsy because of severe painful paresthesia and numbness of lower limbs. The initial neurological clinical and EMG examinations excluded large fiber neuropathy at the inferior extremities in all patients. Three patients were excluded for the remission of symptoms before histological evaluation and one for concomitant mild psychiatric disorder. Therefore, nineteen patients underwent skin biopsy and based on histological findings, were classified as SFN+ or SFN−. The flow chart of the study is shown in Figure 1.

### 3.1. Differences in Biomarkers between SFN+ and SFN− Patients at Follow-Up

Out of the 19 patients who underwent skin biopsy, none had concomitant infections or cancer as well as previous neurological or rheumatic diseases. During neurological or rheumatological symptoms, no patient was positive for anti-SARS-CoV-2 IgM.

**Figure 2:**
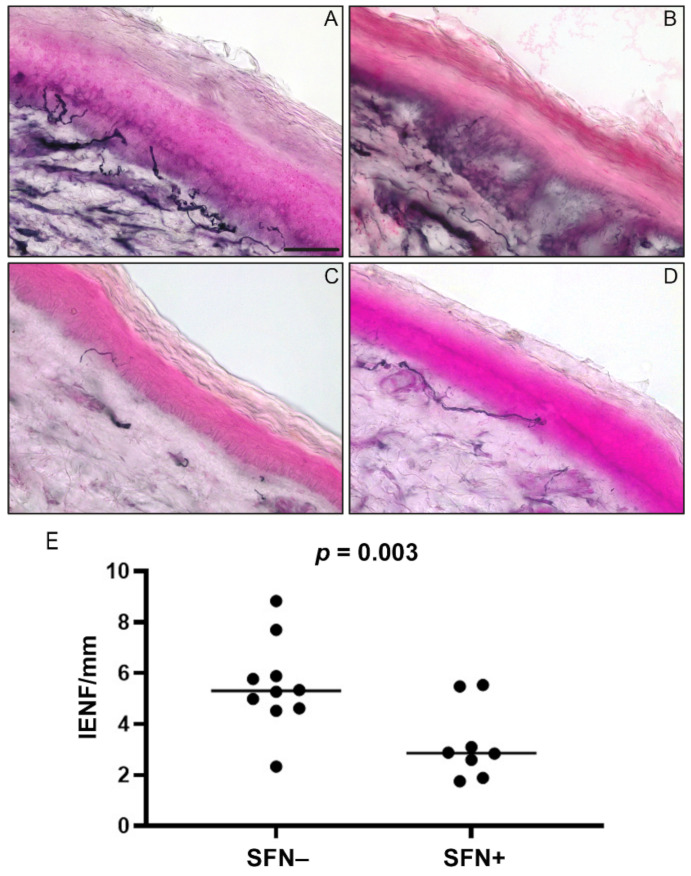
Histological examination of distal leg skin biopsy sections. (**A**–**D**) Representative images of brightfield PGP9.5 immunostaining showing intraepidermal nerve fibers in SFN− (**A**) and SFN+ (**B**–**D**) patients. Images are representative of one SFN− patient (**A**) evolving into SFN+ at six-month follow-up (**B**), and one SFN+ patient at baseline (**C**) and at six-month follow-up (**D**). Scale bar: 50 µm. (**E**) Comparison of intraepidermal nerve fiber density (IENF/mm) between SFN− (*n* = 10) and SFN+ (*n* = 8) patients. SFN, small fiber neuropathy.

Representative histological findings of skin sections immunostained for the nerve fiber marker PGP9.5 are shown in Figure 2A–D.

Seven out of 19 patients were SFN+ at the first skin biopsy (T1, baseline), but only 6 underwent the second histologic evaluation after 6 months (T2) because one patient who encompassed temporal arteritis during the six-month follow-up was excluded (Figure 1). In these 6 patients, SFN+ was confirmed at the second skin biopsy (Figure 2C,D).

Out of 12 SFN− patients at first skin biopsy, 10 did not undergo the second histological examination for remission during three months of follow-up, and 2 repeated a second biopsy after 6 months for persistence and progression of neurological symptoms that revealed a new SFN diagnosis (Figure 1 and Figure 2A,B).

Most of the PC patients (6 out of the 8 (75%) SFN+ patients and 5 out of the 10 (50%) SFN− patients) experienced a paucisymptomatic disease (WHO clinical progression scale 2); only two patients (1 out of the 8 (12.5%) SFN+ patients and 1 out of the 10 (10%) SFN− patients) categorized as WHO clinical progression scale 5 were hospitalized and treated with low flow oxygen therapy. SFN− patients mostly had PCV symptoms (4 out of 10 (40%) patients); only one SNF+ patient developed symptoms after vaccination (Table 1). ANA positivity was very frequent in SFN+ patients (82.5%), but there were no significant differences compared to SFN− patients (Table 1).

The IENF/mm was significantly higher in SFN+ patients than in SFN− (*p* = 0.003) (Figure 2E), without significant differences in age and gender between the two cohorts (Table 1).

OCT and EMG alterations were significantly more common in SFN+ patients, while clinical, serological, lung (HRCT and DLCO abnormalities), and US biomarkers were not different between SFN+ and SFN− patients (Table 1).

### 3.2. Description and Follow-Up of the SFN+ Cases

Table 2 shows the demographic and clinical features of the 8 patients who resulted SFN+ at the second skin biopsy performed after six months (T2), and then were further followed up and clinically assessed at 9 (T3) and 12 months (T4).

At acute onset, fatigue, burning pain, sensitivity disorders (numbness, altered thermal sensation, stocking/glove-like disorder), brain and visual fog, accompanied by a severe motor impairment to stand, walk, and climb stairs, with symmetric involvement at calf and feet, extending to the knees and not present at hands or arms.

All 8 cases satisfied clinical (neuropathic symptoms in the distal extremities and evidence for pinprick or thermal hyposensitivity in areas of neuropathic pain, without significant large fiber nerve conduction abnormalities at EMG) and histological criteria for definite SFN.

All patients had IENF/mm reduction at distal legs and not at proximal thighs. Baseline skin biopsies IENF/mm (median 2.86, IQR 2–4.8) did not change significantly at T2 follow-up (median 2.9, IQR 1.9–3.7; *p* = 0.9 by Mann–Whitney U test), even if decreased in 5 out of 8 (62.5%) patients.

As shown in Table 3, while motor impairment, fatigue, and burning pain significantly decreased also between T3 and T4 (*p* = 0.04, *p* = 0.01, and *p* = 0.04, respectively), brain fog and sensitivity disorders (thermal disarray, numbness, stocking-glove) significantly ameliorated only at T4 (comparison between T1 and T4: *p* = 0.01, *p* = 0.0005, *p* = 0.002, and *p* = 0.0005, respectively; Table 3) after treatment with steroids, pregabalin, DMARDs, and multi-integration (vitamin B6-B12 and D3, homotaurine and phosphatidylserine, folate, alpha lipoic acid; Table 2). Three out of 8 patients (37.5%) were also treated with tapentadol, buprenorphine, and duloxetine.

**Figure 3:**
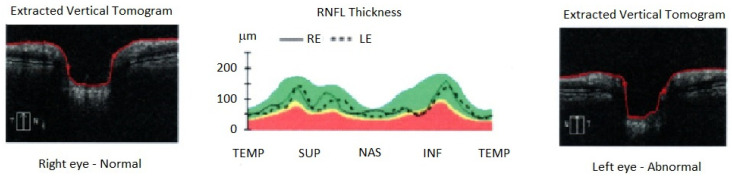
Reduction in thickness (“red zone” of graphic) of peripapillary retinal nerve fiber layer (RNFL) in a patient with post-COVID19-associated small fiber neuropathy presenting visual fog symptoms in the inferior part of the retina (INF) of the left eye (LE) at ocular tomography (OCT), in comparison to superior (SUP), temporal (TEMP), and nasal (NAS) sections. The RNFL is overall lower than in the right normal eye (RE), as demonstrated also by extracted vertical tomography showing an excavation of optic nerve papilla.

On the other side, visual fog did not change during the follow-up, and OCT showed an initial abnormality of retinal pigment epithelium in 4 out of 8 (50%) and a reduction in RNFL thickness in 4 out of 8 (50%) patients at follow-up (Figure 3 and Table 2).

Standard nerve conduction studies revealed, in 6 out of 8 (75%) patients, only isolated abnormalities of Motor Unit Action Potentials (MUAP; short duration and small amplitude), without the spontaneous activity of positive sharp waves and fibrillations, which did not change at follow-up. Only 2 out of 8 (25%) patients presented an associated low isolated positivity for anti-Ro52 and anti-HGMR myositis antibodies, and 2 out of 8 (25%) patients presented an increase in CPK levels. At clinical evaluation, DAS28/ESR significantly changed between T2 and T3 (*p* = 0.04) and at long-term follow-up (T4, *p* = 0.02; Supplementary Figure S1 and Table 3). On the other side, joint US examination showed a change in US pattern yet at six months of follow-up (T2), and a decrease in the involvement from symmetric to asymmetric disease in 7 out of 8 (87.5%) patients (Table 2).

At HRCT, 2 out of 8 (25%) patients had initial lung ground glass, not progressing at imaging follow-up, 5 out of 8 (62.5%) traction bronchiectasis, and 4 out of 8 (50%) subpleural nodules, not progressing at six-month follow-up (T2).

At functional lung tests, 2 out of 8 (25%) patients had a moderate reduction in DLCO, that was stable during the period of observation; only one patient (12.5%) progressed since moderate to severe DLCO reduction at six-month follow-up (T2).

## 4. Discussion

In the present study, we described a case series of acute SFN+ patients with contemporary onset of UA during the PC and PCV period, comparing them to SFN− patients with similar neurological lower limb symptoms.

SFN is a rare disease that affects the somatic and autonomic Aδ myelinated or C unmyelinated fibers of the peripheral nervous system, which prevalently are present inside the skin, but also in other nerves, muscles, and internal organs. Recent evidence showed that SFN symptoms might be present within 4 weeks after COVID-19 infection [25] and might persist in association with brain fog [27], similar to our results.

Although the literature is limited, acute SFN cases were also described after vaccinations, including BioNTech Oxford- ChAdOx1-S COVID-19 [11,28,29], human papillomavirus-varicella, rabies, and Lyme disease vaccines [30,31]. Between vaccine side effects, usually considered self-limiting, the only reported exception was the human papillomavirus vaccine, where there was a more generalized and non-length-dependent pattern, similar to our isolated case of PCV-associated SFN. Unfortunately, other papers on PCV-SFN [28,29] did not report a long-term follow-up of the patients studied, and additional observations are needed including more extensive statistics. The association of SFN with rheumatic diseases, until now, was typically described more frequently as a subtype of fibromyalgia [7] and, less frequently, as an overlap condition with Sjögren’s syndrome [32,33], and lupus [34]. Of note is that, with regards to arthritic diseases, the association of SFN and UA was never described before, except as a complication of anti-TNF-α treatment in rheumatoid arthritis patients who presented a pain distribution often not conforming to the traditional “stocking-and-glove” pattern [35], and generally attributed to the cross-linked immunological reactions to treatment [36].

Amongst biomarkers proposed for SFN in the literature, autoantibodies seemed to be the most promising ones that might be useful in the future to classify the disease phenotype [37]. Moreover, the possible genetic predisposition of SFN is still unknown. However, in our study, we observed only a non-significant mild higher prevalence of ANA in SFN+ compared to SFN− patients (87.5% vs. 50%, respectively), and the prevalence of the studied HLA-haplotypes did not differ between SFN+ and SFN− patients. Nevertheless, considering the relatively small number of patients studied, any possible predisposition needs to be investigated in larger multicenter cohorts. In recent years, there has also been growing interest in the possible implication of genes encoding voltage-gated sodium ion channels in SFN+ patients. Indeed, such channels are transmembrane polypeptides responsible for the generation and conduction of action potentials of excitable cells and are susceptible to mutations due to environmental factors such as viral infections that trigger an overactive immune response [38], as observed mainly in particular cases of polymorphisms. Therefore, future investigations on these peculiar genes might improve our knowledge of the susceptibility to PC- or PCV-associated SFN.

While in our study serological, lung, and joint biomarkers of PC- and PCV-associated SFN+ patients were comparable to those of SFN− patients, similarly to previous observations [27], we also demonstrated that SFN+ patients presented a lower IENF/mm at skin biopsy, independently of demographic parameters, and more frequently MUAP-EMG (75%) and OCT (62.5%) abnormalities compared to SFN− patients.

Even if EMG was used prevalently for SFN diagnosis exclusion and changes of MUAP morphology and shape were described in SFN+ cases only sporadically [19,21], in most of our PC- and PCV-associated SFN+ patients we observed MUAP abnormalities that were clearly different from SFN− patients showing short duration, small amplitude, and polyphasic aspect, without spontaneous activity of positive sharp waves and fibrillations, as previously demonstrated frequently in post-COVID-19 cases [39].

The visual fog was previously underlined in SFN+ [19,40,41], but the OCT-RNFL thickness reduction of the optic nerve was not previously shown in these patients and was described previously only in diabetic subjects [42].

In our study, during a long-term follow-up, the symptoms and the histological and EMG/OCT abnormalities persisted, suggesting that COVID-19 vaccination and infection might trigger a new definitive disease, even if patients seemed partially responders to steroid, immunomodulator, and immunosuppressant drugs, together with multi-integration and pregabalin, after 12 months. Given the lack of specific protocols and recommendations for this rare and emerging disease, our explorative treatment strategy might be useful for future larger cohort studies possibly employing an algorithm for therapy. Indeed, despite initial enthusiasm for SFN immunotherapy [37,43], the benefits of first immunoglobulin therapy are still doubtful and need further validation and testing trials to determine the real efficacy in the future [44]. Current support treatment for SFN consisted of different classes of neuropathic pain medication, including anticonvulsants, antidepressants, opioids, and topical agents; only steroid therapy was more commonly employed in patients with acute SFN with a significant improvement in their clinical symptoms 1–2 weeks later, but with high risk of recurrence after withdrawal [45]. In our study, we used immunomodulators (e.g., hydroxychloroquine, sulfasalazine) for the first time and immunosuppressant (e.g., methotrexate and mycophenolate) drugs that are employed in our daily practice rheumatological protocols to treat PC- and PCV-associated SFN+ patients. In addition, we commissioned, since the first phase of the disease, steroids associated with vitamin B6-B12 and D3, folate, alpha lipoic acid, and pregabalin. In our study, such a therapeutic approach slowly showed good long-term results (after 9 months) on fatigue, muscle motor impairment, burning pain, and arthritis, and later (after 12 months) on brain fog and sensitivity disorders (e.g., thermal disarray disorder, disorders with symptoms of numbness, and stocking-glove disorder). Unfortunately, both the OCT abnormalities and visual fog did not change during the follow-up, but longer follow-up observations on larger cohorts should be considered.

## 5. Conclusions

In the present small case-control monocenter study, we compared the rare manifestations of PC- and PCV-associated SFN+ patients with SFN− patients and found a significantly lower IENF density at skin biopsy, higher MUAP abnormalities and lower RNFL thickness, respectively at EMG and OCT, similarly to other few previous evidences in PC and PCV patients. Autoantibodies and other biomarkers, comprising also genetic factors, seemed not diriment for screening potential risk factors, and, hence, our study confirmed the importance of skin biopsy for a definitive diagnosis of SFN. Future biomarkers for SFN are needed to stratify patients and develop possible targeted treatments other than symptomatic treatments. Our study confirms that SFN triggered by COVID-19 infection or vaccination is a persistent and not self-limiting disease, representing a diagnostic and therapeutic challenge for different specialists. Moreover, the results of our multi-faceted approach suggest that a common accurate, precise, and affordable diagnostic and treatment strategy might in part control pain and sensitive impairment, thus preventing future handicaps of patients and ameliorating their quality of life. As an early diagnosis of SFN has been supposed to possibly control spur axonal repair of peripheral axons in early phases, further studies testing the efficacy of immunomodulatory and immunosuppressant drugs in larger cohorts of patients are essential to improve symptoms in long-term follow-up, and possibly reach a complete disease remission. Finally, an in-depth understanding of SFN pathophysiology is required to explore more targeted treatments.

## Figures and Tables

**Table 1:** Differences in biomarkers between SFN+ and SFN− patients at second skin biopsy.

Clinical and Instrumental Data (Median, IQR or *n*, %)	SFN+ (*n* = 8)	SFN− (*n* = 10)	*p*-Value
Age (years)	55.5 (47–63.2)	54.5 (47–69.2)	0.9 *
Female sex (*n*, %)	5 (62.5%)	8 (80%)	0.4 **
Time between infection or vaccine and “long-COVID19 and long-vaccine” symptoms onset (days)	4 (1–11)	2.5 (1–5.6)	0.9 *
Post-COVID19-vaccine	1/8 (1/8 BNT162b2-mRNA-1273)	4/10 (3/10 BNT162b2-mRNA; 1/10 AZD1222)	0.2 **
IENF/mm	2.8 (2–8)	5.3 (4.6–6.4)	0.003 *
CRP (mg/dL)	0.5 (0.1–1.1)	0.4 (0.1–0.9)	0.03 *
ESR (mm/h)	21.6 (8.3–35)	22.6 (5.5–39)	0.09 *
IL-6 (pg/mL) mean	2.9 (2.1–4.7)	2.9 (2.3–4.2)	0.09 *
ANA speckled >1:160% and titre	7/8 (87.5%) 170 (114–225)	5/10 (50%) 136 (82–190)	0.09 **; 0.2 *
Anti-spike protein antibody BAU Who/mL	799 (212–1387)	869 (265–1473)	0.08 *
Anti-SARS-CoV-2 antibody IgG, AU/mL	33 (9.1–57.6)	43 (2–88)	0.9 *
NK (cells/mcl)	269.4 (144–393)	284 (177–390)	0.7 *
CPK UI/L	87.5 (41–134)	124.5 (81–134)	0.4 *
Aldolase UI/Ml	5.2 (4.4–6.4)	6.2 (4.4–7)	0.6 *
HLA DRB1*11, C*07 positive	5/8; 3/8	5/10; 2/10	0.6 **; 0.4 **
MUAP abnormalities	6/8 (75%)	3/10 (30%)	0.04 **
OCT abnormalities	6/8 (75%)	0/10 (0%)	0.001 **
HRCT abnormalities	2/8 (25%) ground glass; 5/8 (62.5%) bronchiectasis; 4/8 (50%) subpleural fibrotic nodules	1/10 (10%) ground glass; 5/10 (50%) bronchiectasis, 3/10 (30%) subpleural fibrotic nodules	0.6, 0.4, 0.5 **
DLCO abnormalities	3/8 (37.5%)	1/10 (10%)	0.2 **
Synovitis US positive	7 (87.5%)	7 (70%)	0.4 **
Tenosynovitis US positive	6 (75%)	6 (60%)	0.5 **
Pseudo-tenosynovitis US positive	5 (62.5%)	7 (70%)	0.7 **

Values are expressed in median and interquartile (IQR) and percentage; *p*-values calculated by * Mann–Whitney U test and ** Chi-square test. Abbreviations: ANA, antinuclear antibodies; CPK, creatine phosphokinase; CRP, C-reactive protein; ESR, erythrocyte sedimentation rate; HRCT: high resolution computerized tomography; IENF/mm: mean density of intraepidermal nerve fibers; IL-6: interleukin 6; NK: natural killer cells, MUAP: Electromyography Motor Unit Action Potentials; SFN: small fiber neuropathy.

**Table 2:** Demographic and clinical features of the 8 SFN+ patients at second skin biopsy.

SFN+: Age at Onset, PC/PCV, Onset Time	IENF/mm	MUAP, OCT, Joint US and RNFL		Treatments
43 yrs, PC, 15 days,	5.5 (T1); 4 (T2)	MUAP abnormalities (T1 and T2); normal RNFL (T1), reduction in RNFL thickness (T2)	Symmetric SYN+ and TS+ (T1); asymmetric TS+ (T2)	Steroids, vitamin B6–B12 and D3, homotaurine and phosphatidylserine, folate, alpha lipoic acid, pregabalin, HCQ, MTX
61 yrs, PC, 1 day	2.6 (T1); 2.8 (T2)	MUAP abnormalities (T1 and T2); initial dystrophy of RPE and reduction in RNFL thickness (T1 and T2)	Symmetric SYN+, TS+, and PT+ (T1); asymmetric SYN+ and PT+ (T2)	Steroids, vitamin B6-B12 and D3, folate, alpha lipoic acid, pregabalin, HCQ, SLZ, mycophenolate
57 yrs, PCV, 1 day	2.8 (T1); 3 (T2)	MUAP abnormalities (T1 and T2); reduction of RNFL thickness (T1 and T2)	Symmetric SYN+, TS+, and PT+ (T1); asymmetric TS+ (T2)	Steroids, vitamin B6–B12 and D3, folate, alpha lipoic acid, pregabalin, HCQ, mycophenolate
54 yrs, PC, 2 days	7.3 (T1); 3.1 (T2)	MUAP abnormalities (T1 and T2); normal RNFL (T1 and T2)	Symmetric SYN+, TS+, and PT+ (T1); asymmetric TS+ and PT+ (T2)	Steroids, vitamin B6-B12 and D3, folate, alpha lipoic acid, pregabalin, HCQ, sulfasalazine, mycophenolate
45 yrs, PC, 2 days	9.3 (T1); 5.5 (T2)	MUAP abnormalities (T1 and T2); normal RNFL (T1 and T2)	Symmetric SYN+, TS+, and PT+ (T1); asymmetric SYN+, TS+, and PT+ (T2)	Steroids, vitamin B6-B12 and D3, folate, alpha lipoic acid, pregabalin, HCQ, sulfasalazine, mycophenolate
53 yrs, PC, 1 day	2.9 (T1); 2.7 (T2)	Normal MUAP (T1 and T2); initial dystrophy of RPE and normal RNFL (T1 and T2)	Symmetric SYN+, TS+, and PT+ (T1); symmetric SYN+ and PT+ (T2)	Steroids, vitamin B6-B12 and D3, folate, alpha lipoic acid, pregabalin, HCQ
78 yrs, PC, 2 days	1.9 (T1); 1.3 (T2)	MUAP abnormalities, (T1 and T2); initial dystrophy of RPE (T1 and T2) and reduction of RNFL (T2)	Symmetric SYN+, TS+ and PT+ (T1); normal (T2)	Steroids, vitamin B6-B12 and D3, folate, alpha lipoic acid, pregabalin, HCQ
64 yrs, PC, 2 days	1.7 (T1); 1.7 (T2)	Normal MUAP (T1 and T2); normal RNFL (T1 and T2)	Symmetric SYN+, TS+, and PT+ (T1); symmetric SYN+ and PT+ (T2)	Steroids, vitamin B6-B12 and D3, folate, alpha lipoic acid, pregabalin, HCQ, methotrexate

T1: baseline; T2: six-month follow-up. Abbreviations: PC, post-COVID-19; PCV, post-COVID-19 vaccination; IENF/mm: mean density of intraepidermal nerve fibers; MUAP, Motor Unit Action Potentials; RNFL, retinal nerve fiber layer; OCT, optical coherence tomography; RPE, retinal pigment epithelium; SYN, synovitis; TS, tenosynovitis; PT, pseudo-tenosynovitis; HCQ, hydroxychloroquine; SLZ, sulphasalazine; MTX, methotrexate.

**Table 3:** Clinical parameters of SFN+ patients at follow-up.

SFN+ Clinical Parameters Median (IQR)	T1 (Baseline)	T2 (6 Months)	T3 (9 Months)	T4 (12 Months)
DAS28/ESR	5.2 (4.3–5.4) T1–T2 ns	4.8 (3.9–5.3) T2–T3 *p* = 0.04 *	3.4 (3–4.9) T3–T4 ns	2.9 (3.4–4.8) T1–T4 *p* = 0.02 *
Motor impairment	9.5 (7.5–10) T1–T2 ns	8 (5.7–8.5) T2–T3 ns	5 (4–8) T3–T4 *p* = 0.04 *	2 (1.5–5) T1–T4 *p* = 0.0003 *
Fatigue (0–10)	10 (9–10) T1–T2 ns	8.5 (8–10) T2–T3 ns	8 (5–9) T3–T4 *p* = 0.01 *	3 (2–7) T1–T4 *p* = 0.0003 *
Burning pain (0–10)	10 (9–10) T1–T2 *p* = 0.04 *	8 (8–9,7) T2–T3 ns	8 (6–10) T3–T4 *p* = 0.04 *	5 (2–7) T1–T4 *p* = 0.0002 *
Numbness (0–10)	9.5 (9–10) T1–T2 ns	8 (7–9.7) T2–T3 ns	8 (5–10) T3–T4 ns	4 (1–7) T1–T4 *p* = 0.0005 *
Thermal disarray (0–10)	9.5 (8.2–10) T1–T2 ns	8 (6.5–9.5) T2–T3 ns	8 (3–10) T3–T4 ns	5 (2–7) T1–T4 *p* = 0.002 *
Stocking-glove (0–10)	10 (9–10) T1–T2 ns	8 (7.2–9.7) T2–T3 ns	7 (3–8) T3–T4 ns	4 (2–7) T1–T4 *p* = 0.0005 *
Brain fog (0–10)	9 (6–10) T1–T2 ns	8 (7.2–8.7) T2–T3 ns	7 (5–8) T3–T4 ns	4 (2–8) T1–T4 *p* = 0.01 *
Visual fog (0–10)	2.5 (2–3.7) T1–T2 ns	3.5 (3–5) T2–T3 ns	3 (3–5) T3–T4 ns	3 (1–5) T1–T4 ns

Values are expressed in median and interquartile range (IQR); * *p*-values calculated by Mann–Whitney U test. Abbreviations: DAS28/ESR, disease activity score in 28 joints calculated on erythrocyte sedimentation rate levels; SFN, small fiber neuropathy; ns, not significant.

## Data Availability

The original contributions presented in this study are included in the article. Further inquiries can be directed to the relevant author.

## Supplementary Materials

The following supporting information can be downloaded at: https://www.mdpi.com/article/10.3390/jpm14080789/s1, Figure S1: Amelioration of clinical parameters at long-term follow-up. T1 (baseline), T2 (6 months), T3 (9 months), and T4 (12 months). Significance and non-significance (n.s.) of comparisons by the Mann–Whitney U test are indicated in images. (A) DAS28/ESR; (B) brain fog; (C) burning pain; (D) fatigue; (E) thermal disarray; (F) numbness; (G) stocking-glove disorder; and (H) motor impairment.
