# Supplementary material for: Small Fiber Neuropathy Associated with Post-COVID-19 and Post-COVID-19 Vaccination Arthritis: A Rare Post-Infective Syndrome or a New-Onset Disease?
Source: J Pers Med. 2024 Jul 25;14(8):789. doi: 10.3390/jpm14080789 (PMC11355276; doi:10.3390/jpm14080789)
Supplement: Supplementary file 1 [file jpm-14-00789-s001.zip › jpm-3100561-supplementary.pdf]

Figure S1: clinical parameters decrease at long term follow up: T1 baseline, T2 six months, T3 nine months, T4 12 months. Mann Whitney test significance and non-significance (n.s.) of comparison was indicated in images. S2A: DAS28(ESR); S2B: brain fog; S2C: burning pain; S2 D: fatigue; S2 E: thermal disarray; S2 F: numbness; S2 G: stocking-glove disorder; S2H: motor impairment.

S2A-B

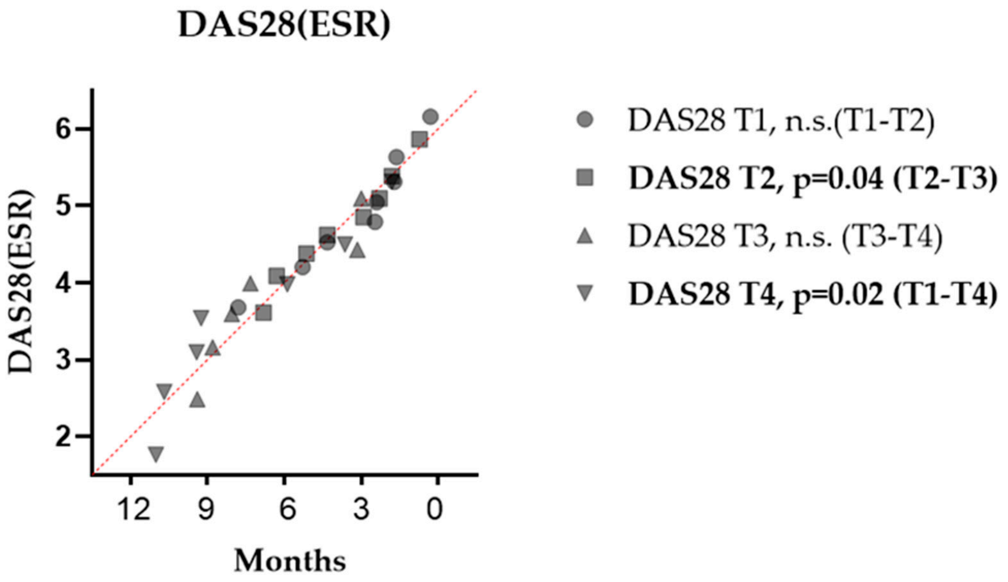

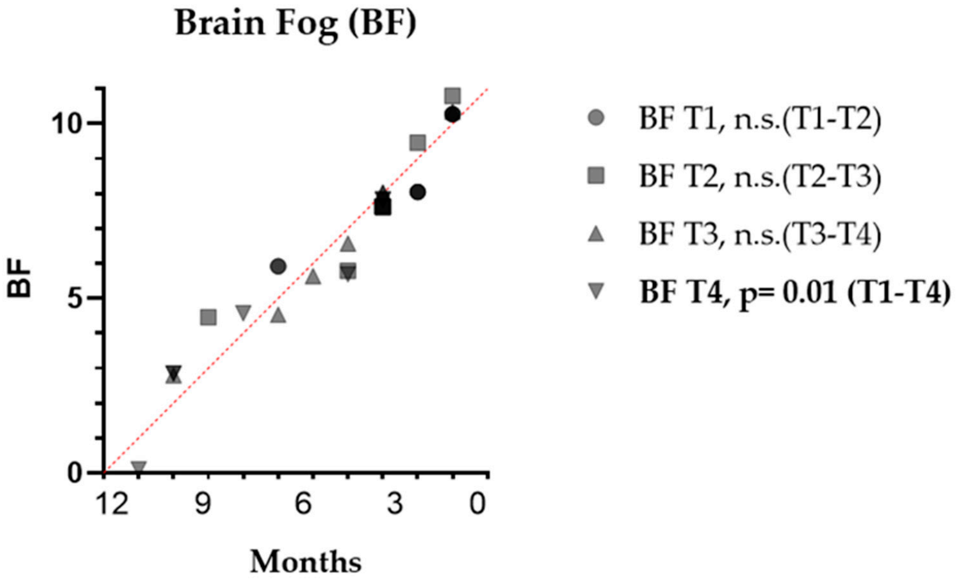

S2C-D.

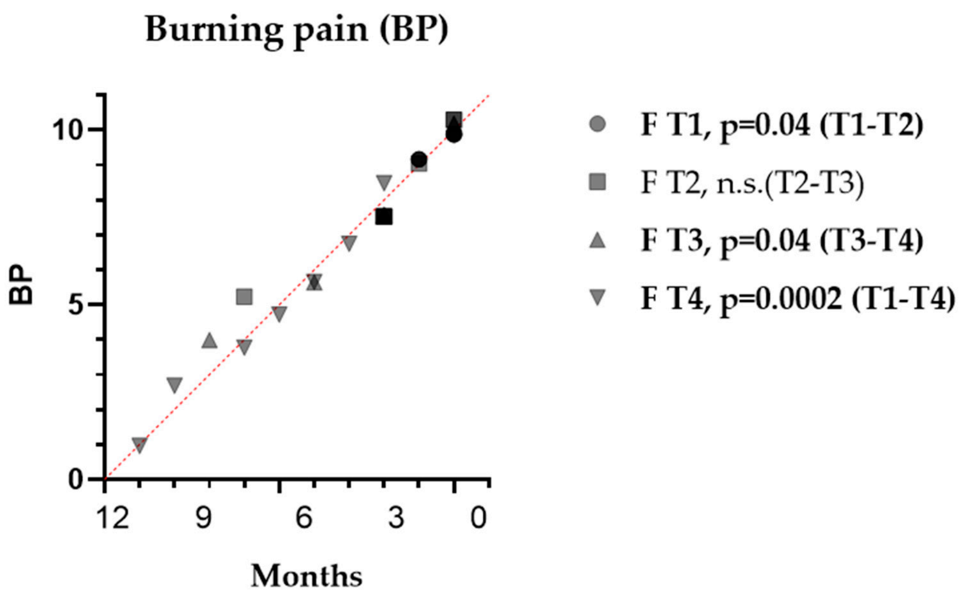
Burning pain (BP)
BP
10
5
0
12
9
6
3
0
Months
F T1, p=0.04 (T1-T2)
F T2, n.s.(T2-T3)
F T3, p=0.04 (T3-T4)
F T4, p=0.0002 (T1-T4)

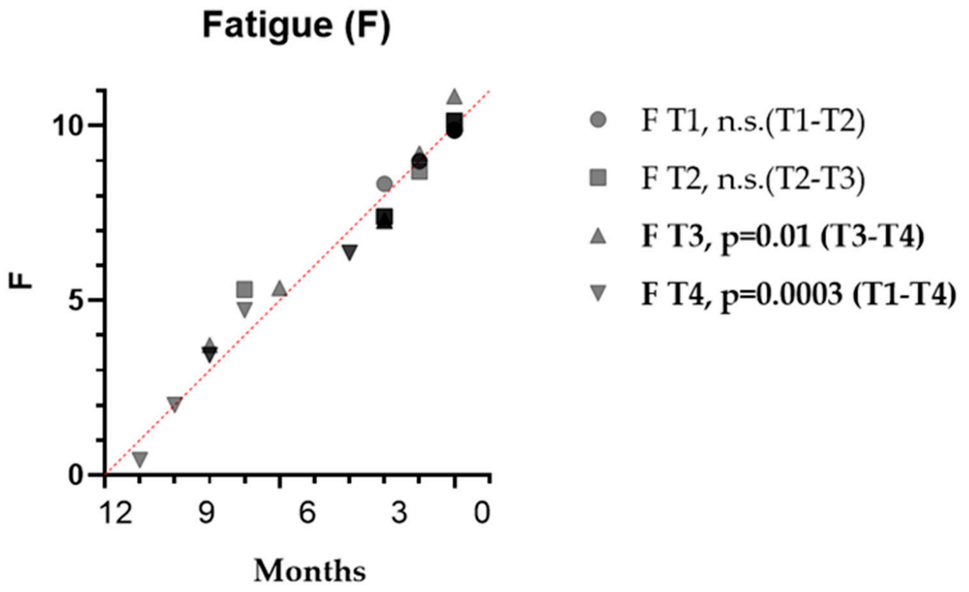
Fatigue (F)
F
10
5
0
12
9
6
3
0
Months
F T1, n.s.(T1-T2)
F T2, n.s.(T2-T3)
F T3, p=0.01 (T3-T4)
F T4, p=0.0003 (T1-T4)

S2 E-F.

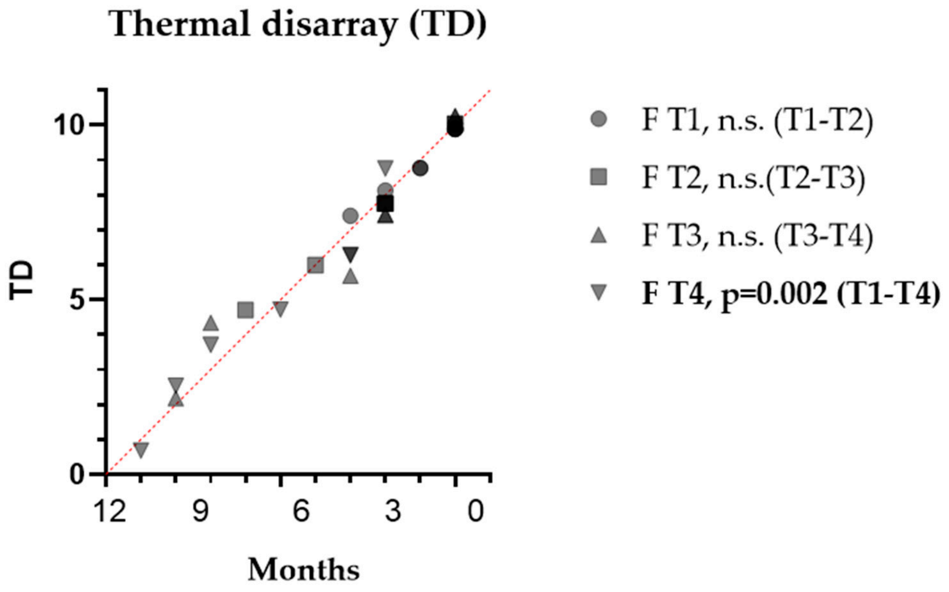
Thermal disarray (TD)
TD
10
5
0
12
9
6
3
0
Months
F T1, n.s. (T1-T2)
F T2, n.s.(T2-T3)
F T3, n.s. (T3-T4)
F T4, p=0.002 (T1-T4)

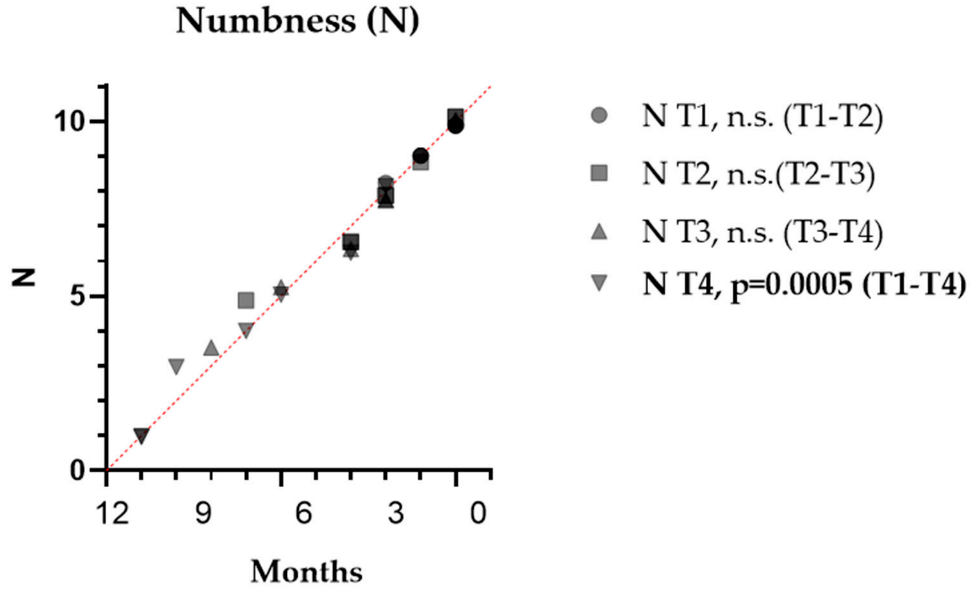
Numbness (N)
N
10
5
0
12
9
6
3
0
Months
N T1, n.s. (T1-T2)
N T2, n.s.(T2-T3)
N T3, n.s. (T3-T4)
N T4, p=0.0005 (T1-T4)

S2 G-H

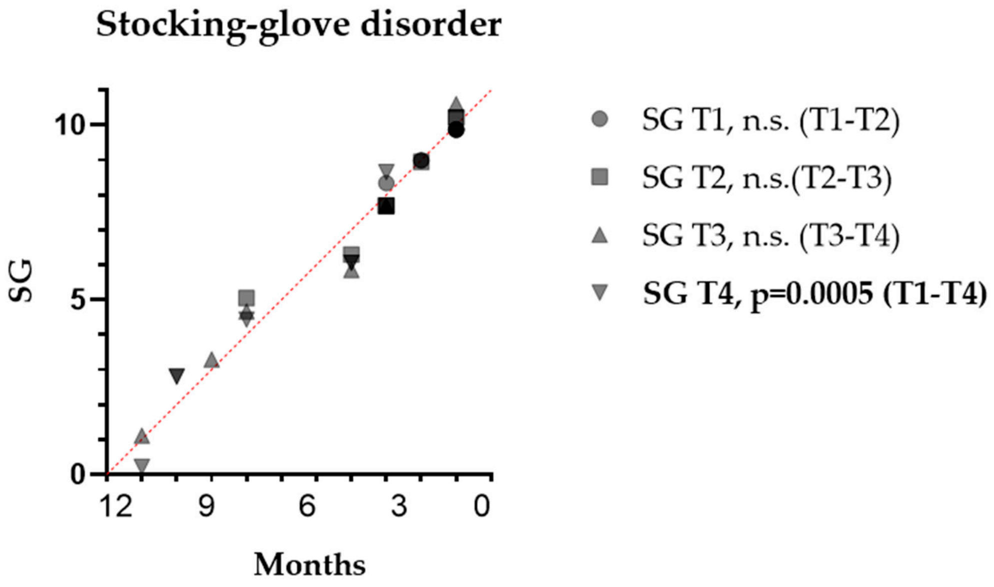
Stocking-glove disorder
SG
10
5
0
12
9
6
3
0
Months
SG T1, n.s. (T1-T2)
SG T2, n.s.(T2-T3)
SG T3, n.s. (T3-T4)
SG T4, p=0.0005 (T1-T4)

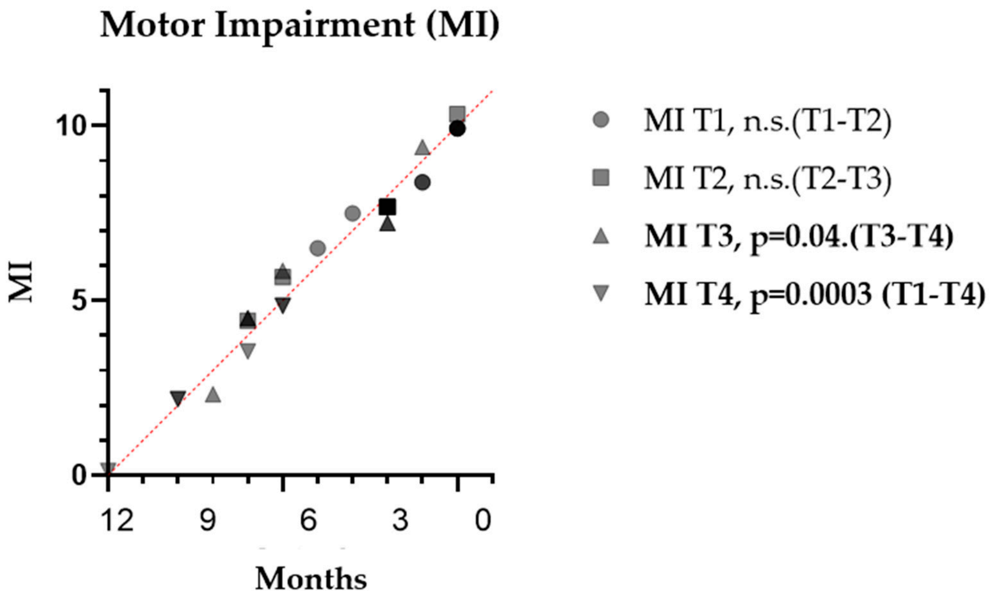
Motor Impairment (MI)
MI
10
5
0
12
9
6
3
0
Months
MI T1, n.s.(T1-T2)
MI T2, n.s.(T2-T3)
MI T3, p=0.04.(T3-T4)
MI T4, p=0.0003 (T1-T4)
